# Systolic characteristics and dynamic changes of the mitral valve in different grades of ischemic mitral regurgitation – insights from 3D transesophageal echocardiography

**DOI:** 10.1186/s12872-018-0819-z

**Published:** 2018-05-10

**Authors:** Caroline Morbach, Diego Bellavia, Stefan Störk, Lissa Sugeng

**Affiliations:** 1 Yale School of Medicine, Section Cardiovascular Medicine, 330 Cedar Street, P.O Box 208017, New Haven, CT 06511 USA; 2 Comprehensive Heart Failure Center and Department of Internal Medicine I, University of Würzburg, Würzburg, Germany; 3 Istituto Mediterraneo per i Trapianti e Terapie ad Alta Specializzazione (IRCCS-ISMETT), Palermo, Italy

**Keywords:** Three-dimensional echocardiography, Mitral valve, Functional regurgitation, Dynamic, Leaflet, Coaptation line, Tenting, Ischemic, Therapeutic approach

## Abstract

**Background:**

Mitral regurgitation in ischemic heart disease (IMR) is a strong predictor of outcome but until now, pathophysiology is not sufficiently understood and treatment is not satisfying. We aimed to systematically evaluate structural and functional mitral valve leaflet and annular characteristics in patients with IMR to determine the differences in geometric and dynamic changes of the MV between significant and mild IMR.

**Methods:**

Thirty-seven patients with IMR (18 mild (m)MR, 19 significant (moderate+severe) (s)MR) and 33 controls underwent TEE. 3D volumes were analyzed using 3D feature-tracking software.

**Results:**

All IMR patients showed a loss of mitral annular motility and non-planarity, whereas mitral annulus dilation and leaflet enlargement occurred in sMR only. Active-posterior-leaflet-area decreased in early systole in all three groups accompanied by an increase in active-anterior-leaflet-area in early systole in controls and mMR but only in late systole in sMR.

**Conclusions:**

In addition to a significant enlargement and loss in motility of the MV annulus, patients with significant IMR showed a spatio-temporal alteration of the mitral valve coaptation line due to a delayed increase in active-anterior-leaflet-area. This abnormality is likely to contribute to IMR severity and is worth the evaluation of becoming a parameter for clinical decision-making. Further, addressing the leaflets aiming to increase the active leaflet-area is a promising therapeutic approach for significant IMR. Additional studies with a larger sample size and post-operative assessment are warranted to further validate our findings and help understand the dynamics of the mitral valve.

## Background

Mitral regurgitation is a strong predictor of cardiac outcome in patients with ischemic heart disease and is associated with higher mortality [1, 2]. The treatment of mitral regurgitation in ischemic heart disease (IMR) has been debated for several decades: Mitral valve (MV) repair is favored due to lower perioperative morbidity and mortality [3, 4], whereas MV replacement if favored by others due to better long-term outcomes and lower IMR recurrence rates [5–7]. A detailed comprehension of the dynamic mitral valve anatomy and function across the cardiac cycle might help to develop and advance novel and more specific treatment options.

IMR has been subject to detailed investigation using three-dimensional (3D) echocardiography and important changes of the MV apparatus have been found. The mitral annulus has been shown to increase in size, flatten its saddle-like shape and loose its dynamic function with higher degrees of IMR [8–19]. Nevertheless, little is known about the changes of the dynamic leaflet motion and function in significant IMR and the differences amongst diverse stages of IMR severity. We therefore aimed to systematically evaluate structural and functional mitral valve leaflet and annular characteristics in patients with IMR to determine the differences in geometric and dynamic changes of the MV between significant and mild IMR.

## Methods

### Study population

We analyzed the stored images of 37 consecutive patients with ischemic heart disease, who had a TEE out of clinical reasons. Inclusion criteria were 1) ischemic heart disease shown by cardiac catheterization, 2) mitral regurgitation of any degree and 3) structurally normal mitral valve in comprehensive echocardiographic evaluation. Exclusion criteria were 1) acute myocardial infarction, 2) contraindications to undergoing TEE, 3) structural mitral valve or subvalvular disease (i.e. degenerative mitral valve disease, prolapse, flail leaflet, cleft, post-endocarditic lesion), 4) significant regurgitation or stenosis of any other cardiac valve, 5) other cardiac disease. IMR severity was determined according to current guidelines [20, 21] and graded into mild (m)MR and significant (s)MR, the latter subsuming moderate and severe IMR. Thirty three subjects without valvular or structural heart disease undergoing TEE for other clinical reasons (i.e. exclusion of endocarditis or search of cardiac source of embolism after ischemic stroke) served as controls.

### Echocardiography

TEE was performed according to the American Society of Echocardiography Guidelines [22] using an x-7 T MTEE probe (Philips Medical Systems, Andover, MA) and standard views were acquired. Additionally, 3D volumes of the mitral valve were recorded. A 4-beat wide-angled acquisition using ECG gating created a full-volume scan of about 90°× 90° volume. Left ventricular ejection fraction (LVEF) was determined by the Simpson’s biplane method. Wall motion abnormalities (WMA) were determined and patients were scored according to their number of by WMA affected LV regions (septal, lateral, inferior, posterior, anterior, apical).

### Offline analysis

**Fig. 1:**
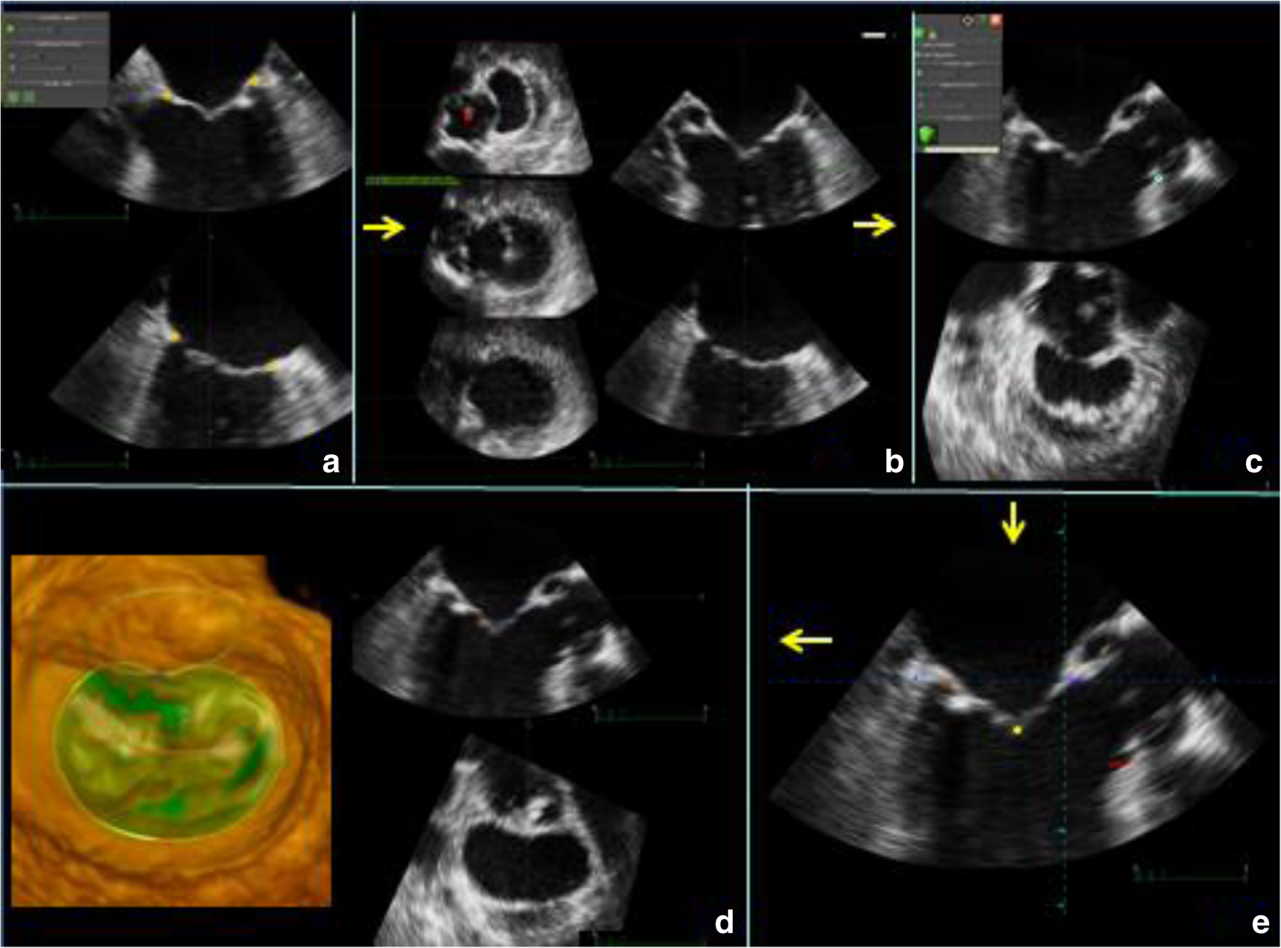
**a** Opening the 3D study, the system displays a four and a two chamber view where the observer has to manually set a total of four landmarks (orange dots) to indicate the mitral valve annulus in two perpendicular planes; **b** as a second step, the aortic valve has to be identified (red dot); **c** subsequently, a long axis view is displayed where the observer has to indicate the apical aortic annulus point (blue dot); **d** finally, the observer has to mark the mitral leaflets coaptation (yellow dot) in the same long axis view; **e** when all landmarks have been set, the program automatically tracks the mitral valve

**Fig. 2:**
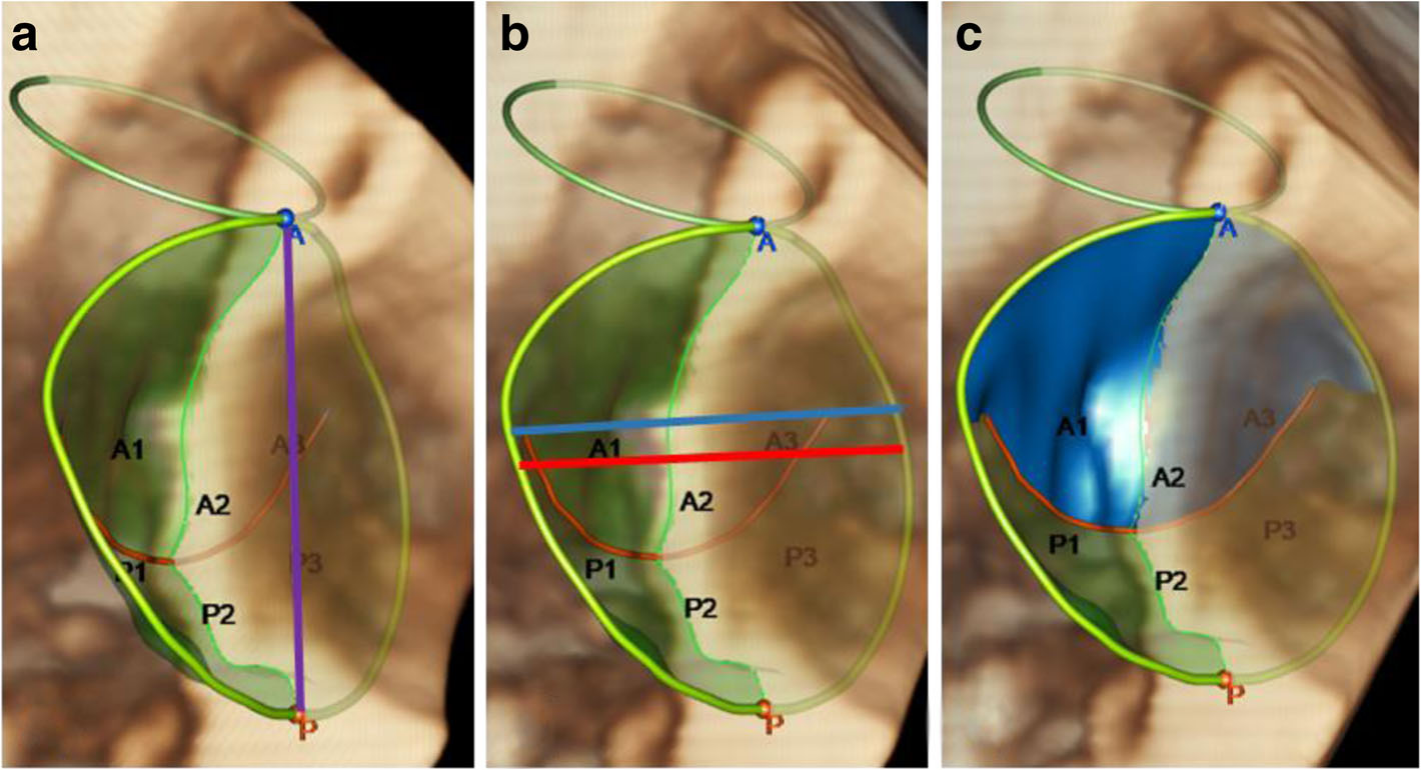
Parameters of the mitral valve analysis. Anterior view of the mitral valve in mid-systole (3D TEE, surface rendering images). The semi-automated analysis of the mitral valve gives the following results: Panel **a**: scheme of the mitral valve including the mid-anterior point (blue dot), the mid-posterior point (red dot), the anterior-posterior diameter as distance between mid-anterior and mid-posterior point (purple line), and the circumference framing the annulus area (yellow circle line). Panel **b**: the anterolateral-posteromedial diameter gives the maximal distance in the horizontal plane (red line), and the intercommissural diameter gives the horizontal diameter at the commissures’ insertion (blue line). Panel **c**: the anterior (blue) and posterior (green) leaflet area is defined as the area between the annulus and the coaptation line (red line) not including the part of the leaflets that form the coaptation zone

**Fig. 3:**
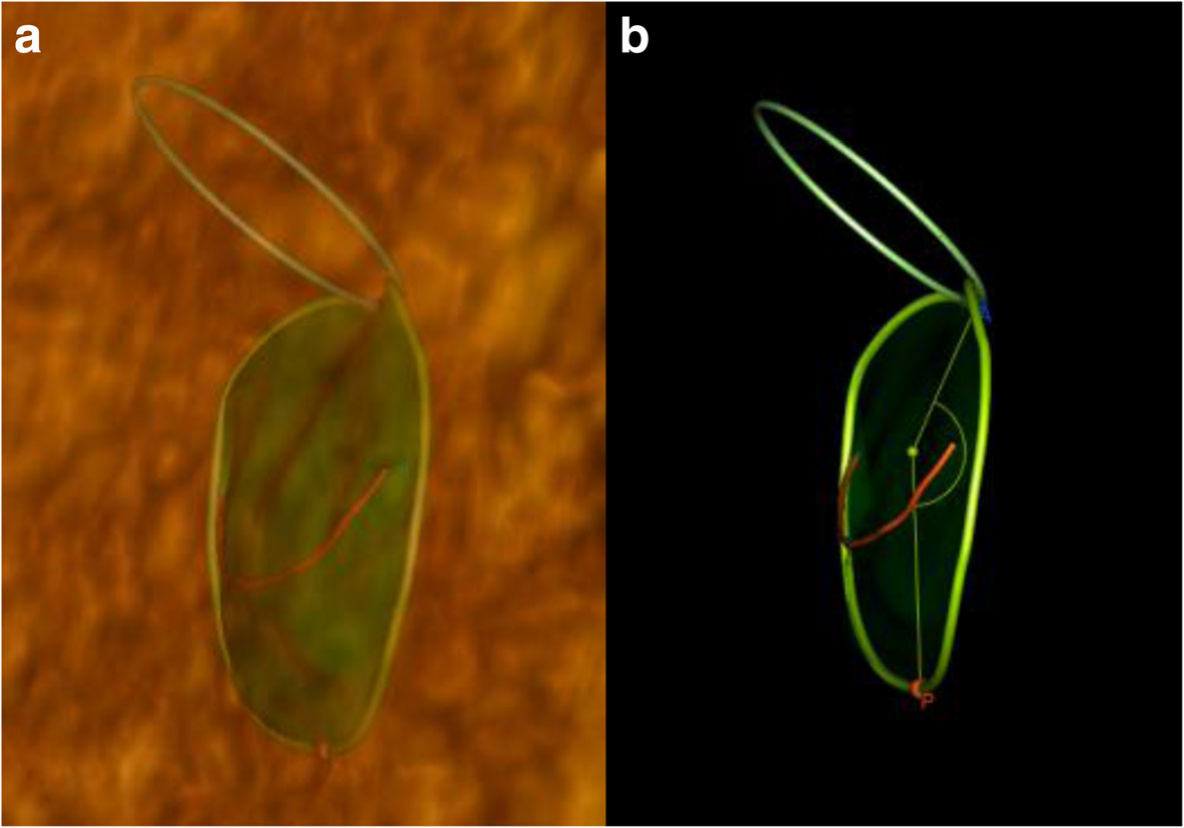
Non-planar angle. Anterior view of the mitral valve in mid-systole: **a** 3D TEE surface rendering. **b** wire frame images. The non-planar angle is assessed as maximal level of “non-planarity” on the line between mid-anterior (blue) and mid-posterior (red) point

Blinded to MR severity, we used custom 3D feature-tracking software (TomTec® Imaging Systems GmbH, Unterschleißheim, Germany) to analyze the datasets. As ECG information is lost when data are exported to the software platform, we identified end-diastole as the first frame when the mitral valve was completely closed and end-systole as the frame preceding mitral valve opening. Initial frame of interest was mid-systole, the mid-frame between end-diastole and end-systole. Mitral annular tracking points were identified in two perpendicular planes in mid-systole. The aortic valve and the apical aortic annulus point were marked and the leaflet coaptation line was defined in a 3-chamber view plane. After defining those landmarks, the program automatically tracked the valve in the initial frame and adjustments of the mitral annulus and the leaflets were performed when necessary (Fig. 1). Subsequently, the program automatically tracked the valve in all frames between end-diastole and end-systole and again, manual adjustments if appropriate. As final step the MV parameters (Figs. 2 and 3) were calculated, saved and exported for further analysis. For quality assurance, a random sample of 10 patients was selected for assessing inter- and intraobserver variability and read in a blinded fashion by an expert in echocardiography once and by another expert twice, more than 4 weeks apart.

### Data analysis

Data analysis was performed using Prism6® (GraphPad, LaJolla, CA, USA). Patient characteristics and echocardiography variables are presented as mean ± standard deviation (SD). Inter- and intra-observer variability are expressed as the absolute difference between two measurements as a percentage of their mean values. For categorical variables differences between groups were assessed using Fishers’s exact test. The difference in WMA score between mMR and sMR was calculated using Mann-Whitney Test. For continuous variables differences between groups were analyzed by one-way analysis of co-variance (ANCOVA), and corrected for multiple testing by the Holm-Sidak method. All variables were assessed for normality using the Shapiro-Wilks test, and non-parametric variables were logarithmically transformed prior to analysis. All tests were two-sided, and *p*-values < 0.05 were considered statistically significant.

## Results

### Subject characteristics

Table 1 shows the characteristics of the three groups. Sex, age and height were not significantly different between the three groups. Patients with mMR had a significantly higher weight and body surface area. Left ventricular ejection fraction was significantly lower and left ventricular end-diastolic diameter larger in both patient groups compared to controls, but there was no difference amongst the IMR groups. Owing to the ischemic etiology, wall motion abnormalities were present in both IMR groups with a preponderance in the lateral and inferior wall sections in sMR. In the mMR and sMR group, one and six patients, respectively, had an IMR jet that was oriented in the posterior or posterolateral direction. All other patients had a central IMR jet.

**Table 1:** Basic characteristics

	Normal	mMR	sMR	P-value		
	*N* = 33	*N* = 18	*N* = 19	Normal vs. mMR	Normal vs. sMR	mMR vs. sMR
Males (%)	42	61	47	ns	ns	ns
Age (years)	58 ± 16	64 ± 11	65 ± 10	ns	ns	ns
Height (cm)	172 ± 12	171 ± 10	166 ± 9	ns	ns	ns
Weight (kg)	82 ± 17	94 ± 20	78 ± 10	0.0394	ns	0.0133
BSA (m^2^)	1.95 ± 0.25	2.05 ± 0.24	1.86 ± 0.15	ns	ns	0.0403
Frame rate (Hz)	23 ± 8	22 ± 4	25 ± 7	ns	ns	ns
LVEF (%)	60 ± 7	43 ± 18	38 ± 17	< 0.0001	< 0.0001	ns
LVEDD (mm)	36 ± 4	44 ± 8	46 ± 10	< 0.0001	< 0.0001	ns
WMA septal (%)	–	50	63	–	–	ns
WMA lateral (%)	–	33	74	–	–	0.0217
WMA inferior (%)	–	50	74	–	–	ns
WMA posterior (%)	–	28	58	–	–	ns
WMA anterior (%)	–	33	63	–	–	ns
WMA apical (%)	–	39	63	–	–	ns

*mMR* mild mitral regurgitation, *sMR* significant mitral regurgitation, vs. versus, *ns* not significant, *LVEF* left ventricular ejection fraction, *LVEDD* left ventricular end-diastolic diameter, *WMA* wall motion abnormalities

Annular displacement (7.5 mm (normal) vs. 5.3 mm (mMR and sMR), *p* = 0.042) and annular displacement velocity (38 mm/s (normal) vs. 25 mm/s (mMR) and 24 mm/s (sMR), *p* <  0.0001), as assessed by 3D analysis, were markedly reduced in both IMR groups with no significant differences between both patients groups. Frame rates were similar amongst all three groups.

### Inter- and intraobserver variability

Coefficients of variation were low (≤6%) for all diameters, annulus circumference, non-planar angle and annular displacement and velocity, whereas coefficients of variation were higher for annulus area and anterior and posterior leaflet area (Table 2).

**Table 2:** Intra- and interobserver variability

MV Parameters	Coefficient of variation (%)	
	Interobserver	Intraobserver
AP diameter	4.5	4.5
AL-PM diameter	5.4	4.3
Non planar angle	1.8	3.1
Annulus circumference	4.4	4.2
Annulus area (3D)	9.0	8.3
Annular displacement	3.2	3.2
Annular displacement velocity	3.4	4.7
Anterior leaflet area	8.3	9.1
Posterior leaflet area	14.6	15.4

*AP* anterior-posterior, *AL-PM* anterolateral-posteromedial, *3D* three dimensional

### Mitral valve structural characteristics in mid-systole

Initial measurements were performed in mid-systole (Table 3). There were no significant differences in diameters, annulus circumference and area, and anterior and posterior leaflet area between controls and mMR, whereas all parameters were significantly increased in sMR compared to controls and mMR. The proportionate increase between controls and sMR in anterior-posterior (AP) diameter (18%) and anterolateral-posteromedial (AL-PM) diameter (18%) as well as in intercommissural diameter (17%) was of similar magnitude.

**Table 3:** Dynamic mitral valve characteristics in systole

MV Parameters	End-diastole			Mid-systole			End-systole		
	normal	mMR	sMR	normal	mMR	sMR	normal	mMR	sMR
AP diameter (cm)	2.60s ± 0.48	2.56 ± 0.48	3.18 ± 0.49^a,b^	2.75 ± 0.46^c^	2.75 ± 0.42^c^	3.25 ± 0.49^a,b,c^	2.78 ± 0.47	2.69 ± 0.41	3.33 ± 0.50^a,b,d^
AL-PM diameter (cm)	3.16 ± 0.50	3.21 ± 0.50	3.78 ± 0.62^a,b^	3.23 ± 0.49^c^	3.28 ± 0.46^c^	3.81 ± 0.61^a,b,c^	3.25 ± 0.49^d^	3.29 ± 0.47	3.83 ± 0.60^a,b,d^
Intercommissural diameter (cm)	3.11 ± 0.49	3.18 ± 0.49	3.72 ± 0.66^a,b^	3.18 ± 0.64^c^	3.25 ± 0.45^c^	3.72 ± 0.65^a,b^	3.20 ± 0.48^d^	3.26 ± 0.45	3.77 ± 0.64^a,b,d^
Annulus circumference (cm)	9.85 ± 1.54	9.59 ± 1.47	11.69 ± 1.99^a,b^	10.14 ± 1.47^c^	9.81 ± 1.29^c^	11.75 ± 1.92^a,b^	10.19 ± 1.49	9.85 ± 1.29	11.86 ± 1.94^a,b,d^
Annulus area 3D (cm^2^)	7.23 ± 2.29	6.95 ± 2.11	10.40 ± 3.72^a,b^	7.71 ± 2.23^c^	7.27 ± 1.91^c^	10.56 ± 3.67^a,b^	7.82 ± 2.27	7.36 ± 1.91	10.82 ± 3.73^a,b,d^
Anterior leaflet area (cm^2^)	4.65 ± 1.29	4.69 ± 1.56	7.23 ± 2.79^a,b^	4.82 ± 1.32^c^	4.75 ± 1.43	7.21 ± 2.78^a,b^	5.00 ± 1.44^d^	4.85 ± 1.42	7.44 ± 2.91^a,b,d^
Posterior leaflet area (cm^2^)	3.78 ± 1.65	3.51 ± 1.33	5.36 ± 0.82 ^a,b^	3.66 ± 1.51	3.40 ± 1.24	4.88 ± 1.94 ^a,b,c^	3.79 ± 1.53	3.46 ± 1.22	5.01 ± 2.15^a,b^
Non planar angle (°)	143 ± 11	155 ± 10^a^	157 ± 10^a^	143 ± 11	154 ± 11^a^	157 ± 10^a^	144 ± 11^d^	155 ± 11^a,d^	160 ± 9^a,d^
Tenting volume (cm^3^)	1.62 ± 0.96	1.82 ± 1.03	3.78 ± 2.13^a,b^	1.05 ± 0.89^c^	1.42 ± 0.82^c^	2.98 ± 1.76^a,b,c^	1.33 ± 1.12	1.50 ± 0.82	3.30 ± 2.15^a,b^
Tenting height (mm)	6.86 ± 2.17	6.39 ± 1.75	9.28 ± 2.10^a,b^	5.26 ± 1.80^c^	5.42 ± 1.82^c^	7.74 ± 2.25^a,b,c^	5.68 ± 2.14	5.39 ± 1.41	7.80 ± 2.56^a,b^

*AP* anterior-posterior, *AL-PM* anterolateral-posteromedial, *mMR* mild mitral regurgitation, *sMR* significant mitral regurgitation, *3D* three dimensional

^a^Significant difference compared to normal in the same phase of cardiac cycle (*p* < 0.05)

^b^Significant difference compared to mild MR in the same phase of cardiac cycle (*p* < 0.05)

^c^Significant difference compared to end-diastolic value in the same patient group (*p* < 0.05)

^d^Significant difference compared to mid-systolic value in the same patient group (*p* < 0.05)

The non-planar angle was significantly flattened in both IMR groups compared to controls with no difference between mMR and sMR patients.

The above detailed mid-systolic differences in MV characteristics between IMR groups and controls were present in end-diastole and end-systole, too (Table 3).

### Mitral valve dynamics in systole

**Fig. 4:**
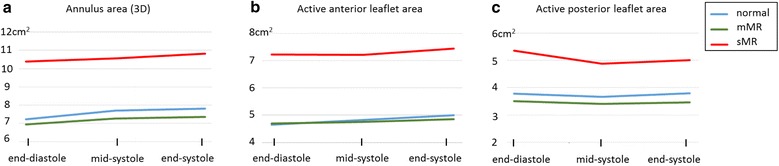
Intrasystolic changes in **a** mitral valve annulus area as well as in **b** active anterior leaflet area and **c** active posterior leaflet area in patients with ischemic heart disease and functional mild (mMR) and significant (sMR = moderate and severe) mitral regurgitation compared to normal controls

In controls, we observed an increase in AP diameter, AL-PM diameter, intercommissural diameter, annulus circumference and annulus area which primarily occurred in early systole (Table 3, Fig. 4). Furthermore, the anterior leaflet area increased homogenously during systole whereas the posterior leaflet area showed a slight but non-significant decrease in early systole (Table 3, Fig. 4).

The widening of the mitral annulus in early systole occurred in mMR as well, but it occurred later in sMR, which was associated with an increase in anterior leaflet area. A decrease in posterior leaflet area in early systole was seen in all three groups but reached significant values in sMR only.

Leaflet tenting volume and height decreased significantly in early systole in all three groups with a slight but insignificant increase in late systole. This was accompanied by flattening of the non-planar angle, which was most obvious in the sMR group.

## Discussion

To our knowledge, this is the first study reporting on the structural and functional dynamic modifications of the mitral valve in different grades of IMR, tracked from end-diastole to end-systole, and assessed by high resolution 3D TEE. The main findings of the present investigation were: 1) as compared to controls or patients with mild valve regurgitation, patients with significant IMR have a bidirectional dilated mitral annulus as well as an increase of the active area of both valve leaflets considered in absolute terms. Furthermore, this group of patients shows 2) loss of the normal mitral non-planar saddle shape, and 3) pronounced reduction of the active-posterior-leaflet-area in early systole as well as 4) delayed dynamic augmentation of the active-anterior-leaflet-area.

We observed 37 patients with IMR and subdivided them by IMR severity. We pooled patients with moderate and severe IMR into one group (i.e., sMR) since it is this group for which mitral valve surgery in combination with coronary artery bypass surgery would be considered [23, 24]. By contrast, in subjects with mMR watchful waiting is advised. Both patient groups had a moderately decreased LVEF with similar LV dimensions and alteration of LV function. However, the frequency of wall motion abnormalities was higher in sMR with a preponderance of lateral LV wall hypokinesis and a posteriorly oriented regurgitation jet.

In our study, intra- and interobserver variability for one-dimensional measurements like annular diameters and circumference was excellent and was similar compared to other studies using semi-automated analysis software [9, 10, 25, 26]. The variability was higher for squared values like annulus and valve areas, where minimal measurement differences induce considerable variation.

### Mitral valve annulus

Patients with mMR showed no significant differences in anatomical annular parameters (diameters, area and circumference) compared to controls, whereas these parameters were significantly larger in sMR. Applying the Pringle Principle, the significant increase in annular circumference and area in absence of difference in planarity in sMR indicates a real dilation of the mitral annulus, not only a change in shape. We further observed smaller absolute and relative intra-systolic changes in annulus area in both IMR groups as correlate for the mitral annulus’ loss of motility, its ability to modify its shape throughout the cardiac cycle [9, 11, 13, 27], involving a flattening of the mitral annulus’ non-planarity.

The dilation of the mitral annulus confirms reports of previous studies [9, 11, 13, 28] and is the basis of standard surgical treatment of functional MR using reduction annuloplasty with an undersized ring [2, 7]. Nevertheless, this treatment alone is not satisfying with persistently high recurrence rates [2, 5]. This brings forth the question, if, although imaging does not show any of the known abnormalities from degenerative valve disease, the leaflets are altered anyway and therefore significantly contribute to IMR severity and might be a target for successful treatment.

### Mitral valve leaflets

Anterior and posterior leaflet areas were significantly larger in sMR compared to controls and mMR. This might be caused by mitral annular enlargement with subsequent augmentation of the leaflets’ circumference but leaflet enlargement also occurs as response to hemodynamic stress and chronic tethering [12, 13, 29]. Despite leaflet augmentation, the leaflets’ adaptation to the new situation does not seem to be sufficient in patients with sMR, and impaired leaflet adaptation rather than the increase in LV and MV size per se seems to cause IMR [30, 31].

The active-posterior-leaflet-area (measured in the closed MV from the annulus to the coaptation line) showed a decrease in early systole, non-significant in controls and mMR but significant in sMR, most probably due to tethering. In return, controls had a significant and homogenous increase in active-anterior-leaflet-area throughout systole, which occurred only in late systole in sMR. This systolic relocation of the coaptation zone with a posterior movement of the coaptation line in competent MVs implies an “anterior leaflet reserve” to compensate for the posterior movement [32, 33]. The MV is able to maintain competency until there is inadequate anterior leaflet coaptation length [33]. Though IMR is believed to be a primary response to ventricular remodeling, compromised anterior leaflet reserve or failure to increase the anterior leaflet area in sMR - as seen in our study - might be a key aspect in the development of moderate to severe mitral regurgitation in ischemic heart disease.

On the one hand, this finding should trigger the proceeding in the development of novel treatment options like surgical elongation of the anterior mitral leaflet [34] or application of neo-chordae in the presence of “pseudoprolapse” of the anterior leaflet [35] as addendum to conventional annuloplasty [36–39]. On the other hand, this finding seems worth the evaluation as a parameter for clinical decision making regarding the optimal treatment time point and treatment option in larger patient cohorts to achieve the best patient’s outcome.

### Limitations

Owing to the study design, we had no consistent information on the onset of coronary artery disease, i.e. the duration of the disease and the dynamic development of IMR and IMR severity. Further, not all patients had a recent angiogram, however, we were able to assess wall motion abnormalities as a surrogate of coronary artery disease in the TEE images. Lastly, the intra- and inter- observer variability for “squared” parameters, which could be an important limitation for common and easily practical application of the software, was higher when compared to “simple” values but still in the range of previous reports [25].

## Conclusions

In addition to a significant enlargement and loss in motility of the MV annulus, patients with significant IMR showed a spatio-temporal alteration of the mitral valve coaptation line due to a delayed increase in active-anterior-leaflet-area. This abnormality is likely to contribute to IMR severity and is worth the evaluation of becoming a parameter for clinical decision-making. Further, addressing the leaflets aiming to increase the active leaflet-area is a promising therapeutic approach for significant IMR. Our observations are based on a small but well characterized patient sample and should therefore be regarded hypothesis-generating; studies with a larger sample size and post-operative assessment are warranted to further validate our findings, explore their pathophysiologic etiology, and help understand the dynamics of the mitral valve.

## Abbreviations

3D: Three-dimensional
AL-PM: Anterolateral-posteromedial
AP: Anterior-posterior
IMR: Ischemic mitral regurgitation
LV: Left ventricle
LVEF: Left ventricular ejection fraction
mMR: Mild mitral regurgitation
MV: Mitral valve
SD: Standard deviation
sMR: Significant mitral regurgitation
TEE: Transesophageal echocardiography
WMA: Wall motion abnormalities

## References

[1] Grigioni F, Enriquez-Sarano M, Zehr KJ, Bailey KR, Tajik AJ (2001). Ischemic mitral regurgitation: long-term outcome and prognostic implications with quantitative Doppler assessment. Circulation.

[2] Timek TA, Miller DC (2011). Another multidisciplinary look at ischemic mitral regurgitation. Semin Thorac Cardiovasc Surg.

[3] Gillinov AM, Wierup PN, Blackstone EH, Bishay ES, Cosgrove DM, White J (2001). Is repair preferable to replacement for ischemic mitral regurgitation?. J Thorac Cardiovasc Surg.

[4] Grossi EA, Goldberg JD, LaPietra A, Ye X, Zakow P, Sussman M (2001). Ischemic mitral valve reconstruction and replacement: comparison of long-term survival and complications. J Thorac Cardiovasc Surg.

[5] LaPar DJ, Acker MA, Gelijns AC, Kron IL (2015). Repair or replace for severe ischemic mitral regurgitation: prospective randomized multicenter data. Ann Cardiothorac Surg.

[6] Glower DD (2012). Surgical approaches to mitral regurgitation. J Am Coll Cardiol.

[7] Acker MA, Parides MK, Perrault LP, Moskowitz AJ, Gelijns AC, Voisine P (2014). Mitral-valve repair versus replacement for severe ischemic mitral regurgitation. N Engl J Med.

[8] Saito K, Okura H, Watanabe N, Obase K, Tamada T, Koyama T (2012). Influence of chronic tethering of the mitral valve on mitral leaflet size and coaptation in functional mitral regurgitation. JACC Cardiovasc Imaging.

[9] Daimon M, Saracino G, Fukuda S, Koyama Y, Kwan J, Song JM (2010). Dynamic change of mitral annular geometry and motion in ischemic mitral regurgitation assessed by a computerized 3D echo method. Echocardiography..

[10] Daimon M, Saracino G, Gillinov AM, Koyama Y, Fukuda S, Kwan J (2008). Local dysfunction and asymmetrical deformation of mitral annular geometry in ischemic mitral regurgitation: a novel computerized 3D echocardiographic analysis. Echocardiography.

[11] Veronesi F, Corsi C, Sugeng L, Caiani EG, Weinert L, Mor-Avi V (2008). Quantification of mitral apparatus dynamics in functional and ischemic mitral regurgitation using real-time 3-dimensional echocardiography. J Am Soc Echocardiogr.

[12] Sprouse C, Mukherjee R, Burlina P (2013). Mitral valve closure prediction with 3-D personalized anatomical models and anisotropic hyperelastic tissue assumptions. IEEE Trans Biomed Eng.

[13] Little SH, Ben Zekry S, Lawrie GM, Zoghbi WA (2010). Dynamic annular geometry and function in patients with mitral regurgitation: insight from three-dimensional annular tracking. J Am Soc Echocardiogr.

[14] Kwan J, Shiota T, Agler DA, Popovic ZB, Qin JX, Gillinov MA (2003). Geometric differences of the mitral apparatus between ischemic and dilated cardiomyopathy with significant mitral regurgitation: real-time three-dimensional echocardiography study. Circulation.

[15] Timek TA, Lai DT, Tibayan F, Liang D, Daughters GT, Dagum P (2003). Ischemia in three left ventricular regions: insights into the pathogenesis of acute ischemic mitral regurgitation. J Thorac Cardiovasc Surg.

[16] Gorman JH, Gorman RC, Jackson BM, Hiramatsu Y, Gikakis N, Kelley ST (1997). Distortions of the mitral valve in acute ischemic mitral regurgitation. Ann Thorac Surg.

[17] Watanabe N, Ogasawara Y, Yamaura Y, Kawamoto T, Toyota E, Akasaka T (2005). Quantitation of mitral valve tenting in ischemic mitral regurgitation by transthoracic real-time three-dimensional echocardiography. J Am Coll Cardiol.

[18] Watanabe N, Ogasawara Y, Yamaura Y, Yamamoto K, Wada N, Kawamoto T (2006). Geometric differences of the mitral valve tenting between anterior and inferior myocardial infarction with significant ischemic mitral regurgitation: quantitation by novel software system with transthoracic real-time three-dimensional echocardiography. J Am Soc Echocardiogr.

[19] Nguyen TC, Itoh A, Carlhall CJ, Bothe W, Timek TA, Ennis DB (2008). The effect of pure mitral regurgitation on mitral annular geometry and three-dimensional saddle shape. J Thorac Cardiovasc Surg.

[20] Nishimura RA, Otto CM, Bonow RO, Carabello BA, Erwin JP, Guyton RA (2014). 2014 AHA/ACC guideline for the management of patients with valvular heart disease: executive summary: a report of the American College of Cardiology/American Heart Association task force on practice guidelines. J Am Coll Cardiol.

[21] Mascherbauer J, Rosenhek R, Bittner B, Binder J, Simon P, Maurer G (2005). Doppler echocardiographic assessment of valvular regurgitation severity by measurement of the vena contracta: an in vitro validation study. J Am Soc Echocardiogr.

[22] Hahn RT, Abraham T, Adams MS, Bruce CJ, Glas KE, Lang RM (2013). Guidelines for performing a comprehensive transesophageal echocardiographic examination: recommendations from the American Society of Echocardiography and the Society of Cardiovascular Anesthesiologists. J Am Soc Echocardiogr.

[23] Chan KM, Punjabi PP, Flather M, Wage R, Symmonds K, Roussin I (2012). Coronary artery bypass surgery with or without mitral valve annuloplasty in moderate functional ischemic mitral regurgitation: final results of the randomized ischemic mitral evaluation (RIME) trial. Circulation.

[24] Daimon M, Fukuda S, Adams DH, McCarthy PM, Gillinov AM, Carpentier A (2006). Mitral valve repair with Carpentier-McCarthy-Adams IMR ETlogix annuloplasty ring for ischemic mitral regurgitation: early echocardiographic results from a multi-center study. Circulation.

[25] Veronesi F, Corsi C, Sugeng L, Mor-Avi V, Caiani EG, Weinert L (2009). A study of functional anatomy of aortic-mitral valve coupling using 3D matrix transesophageal echocardiography. Circ Cardiovasc Imaging..

[26] Mihaila S, Muraru D, Miglioranza MH, Piasentini E, Peluso D, Cucchini U (2015). Normal mitral annulus dynamics and its relationships with left ventricular and left atrial function. Int J Cardiovasc Imaging.

[27] Mihalatos DG, Joseph S, Gopal A, Bercow N, Toole R, Passick M (2007). Mitral annular remodeling with varying degrees and mechanisms of chronic mitral regurgitation. J Am Soc Echocardiogr.

[28] Bartels K, Thiele RH, Phillips-Bute B, Glower DD, Swaminathan M, Kisslo J (2014). Dynamic indices of mitral valve function using perioperative three-dimensional transesophageal echocardiography. J Cardiothorac Vasc Anesth.

[29] Dal-Bianco JP, Aikawa E, Bischoff J, Guerrero JL, Handschumacher MD, Sullivan S (2009). Active adaptation of the tethered mitral valve: insights into a compensatory mechanism for functional mitral regurgitation. Circulation.

[30] Beaudoin J, Handschumacher MD, Zeng X, Hung J, Morris EL, Levine RA (2013). Mitral valve enlargement in chronic aortic regurgitation as a compensatory mechanism to prevent functional mitral regurgitation in the dilated left ventricle. J Am Coll Cardiol.

[31] Beaudoin J, Thai WE, Wai B, Handschumacher MD, Levine RA, Truong QA (2013). Assessment of mitral valve adaptation with gated cardiac computed tomography: validation with three-dimensional echocardiography and mechanistic insight to functional mitral regurgitation. Circ Cardiovasc Imaging.

[32] Zeng X, Nunes MC, Dent J, Gillam L, Mathew JP, Gammie JS (2014). Asymmetric versus symmetric tethering patterns in ischemic mitral regurgitation: geometric differences from three-dimensional transesophageal echocardiography. J Am Soc Echocardiogr.

[33] Gogoladze G, Dellis SL, Donnino R, Ribakove G, Greenhouse DG, Galloway A (2010). Analysis of the mitral coaptation zone in normal and functional regurgitant valves. Ann Thorac Surg.

[34] Rabbah JP, Siefert AW, Bolling SF, Yoganathan AP (2014). Mitral valve annuloplasty and anterior leaflet augmentation for functional ischemic mitral regurgitation: quantitative comparison of coaptation and subvalvular tethering. J Thorac Cardiovasc Surg.

[35] Hashim SW, Youssef SJ, Ayyash B, Rousou AJ, Ragnarsson S, Collazo S (2012). Pseudoprolapse of the anterior leaflet in chronic ischemic mitral regurgitation: identification and repair. J Thorac Cardiovasc Surg.

[36] Rendon F, Aramendi JI, Rodrigo D, Baraldi C, Martinez P (2002). Patch enlargement of the posterior mitral leaflet in ischemic regurgitation. Asian Cardiovasc Thorac Ann.

[37] Langer F, Rodriguez F, Cheng A, Ortiz S, Nguyen TC, Zasio MK (2006). Posterior mitral leaflet extension: an adjunctive repair option for ischemic mitral regurgitation?. J Thorac Cardiovasc Surg.

[38] Dobre M, Koul B, Rojer A (2000). Anatomic and physiologic correction of the restricted posterior mitral leaflet motion in chronic ischemic mitral regurgitation. J Thorac Cardiovasc Surg.

[39] de Varennes B, Chaturvedi R, Sidhu S, Cote AV, Shan WL, Goyer C (2009). Initial results of posterior leaflet extension for severe type IIIb ischemic mitral regurgitation. Circulation.

